# Spindle Cell Tumors with a *GAB1::ABL1* Fusion—Another Member of Protein-Kinase-Related Soft Tissue Neoplasms: An (Epi)Genetic Study of Six Cases with Benign Behavior

**DOI:** 10.3390/ijms27188099

**Published:** 2026-09-11

**Authors:** L. S. Hiemcke-Jiwa, P. Múdry, E. Rullo, B. Koopman, A. H. G. Cleven, J. M. van Gorp, M. M. van Noesel, R. A. Schoot, K. Polášková, M. L. Ooft, N. Veccia, R. Alaggio, S. Barresi, S. Patrizi, E. Miele, S. van Helvert, L. A. Kester, U. Flucke

**Affiliations:** 1 Princess Maxima Center for Pediatric Oncology, 3584 CS Utrecht, The Netherlands; 2 Department of Pathology, University Medical Center Utrecht, 3584 CX Utrecht, The Netherlands; 3 Department of Pediatric Oncology, University Hospital Brno and Faculty of Medicine, Masaryk University, 66263 Brno, Czech Republic; 4 Pathology Unit, Department of Laboratories, Bambino Gesù Children’s Hospital, IRCCS, 00165 Rome, Italy; 5 Department of Pathology, University Medical Center Groningen, 9713 GZ Groningen, The Netherlands; 6 Department of Pathology, Location Antonius Hospital, Pathology-DNA, 3435 CM Nieuwegein, The Netherlands; 7 Department of Pathology, Location Rijnstate Hospital, Pathology-DNA, 6815 AD Arnhem, The Netherlands; 8 King’s College Hospitals, NHS Foundation Trust, London SE5 9RS, UK; 9 Pathology Unit, Department of Oncology and Specialist Medicines, Azienda Ospedaliera San Camillo Forlanini, 00152 Rome, Italy; 10 Onco-Hematology, Cell Therapy, Gene Therapies and Hemopoietic Transplant, Bambino Gesù Children’s Hospital, IRCCS, 00165 Rome, Italy; 11 UniCamillus-Saint Camillus International University of Health Sciences, 00131 Rome, Italy; 12 Department of Pathology, Radboud Institute for Medical Innovation, Radboud University Medical Center Nijmegen, 6525 GA Nijmegen, The Netherlands

**Keywords:** *GAB1::ABL1*, fusion gene, tyrosine kinase, protein kinase, protein kinase, soft tissue tumors, mesenchymal tumors, DNA methylation profiling

## Abstract

Mesenchymal neoplasms driven by protein kinases represent an increasing group with a broad clinicopathologic and genetic spectrum. Recently, tumors harboring a *GAB1::ABL1* fusion have been reported, resulting in constitutive activation of the non-receptor tyrosine kinase ABL1. We describe six cases (one previously reported) investigated by fusion gene analysis and DNA-methylation profiling. Tumors were from three males and three females aged 7–71 years and arose in the neck, axilla, lower back, thigh, and digits. Lesions were excised. Available follow-up was uneventful. Neoplasms were circumscribed but unencapsulated, with infiltration of the surrounding tissue, and characterized by haphazardly arranged monomorphous spindle cells. There was a fibromyxoid stroma and staghorn-like vessels were present. Immunohistochemically, expression of CD34 (3/6), S100 (1/6), EMA (2/6), and GLUT1 (3/3) was observed. All cases harbored a *GAB1::ABL1* fusion. RNA expression profile (n = 2) showed high-confidence similarity to dermatofibrosarcoma protuberans (DFSP). DNA-methylation profiling demonstrated that all cases clustered together in close proximity to DFSP. Application of the latest Heidelberg methylation sarcoma classifier did not confidentially classify these neoplasms. However, four showed low-confidence matches to *NTRK*-rearranged spindle cell neoplasms. *GAB1::ABL1* spindle cell neoplasms represent a distinct member of the family of kinase-driven mesenchymal tumors with an indolent clinical course, while the activating *ABL1* fusion raises the possibility of targeted therapy in selected cases.

## 1. Introduction

With the increased use of advanced molecular techniques, the detection of genetic alterations of tumors is rapidly expanding including a group of mesenchymal lesions with changes in genes encoding for protein kinases (PKs). The involved PKs are mainly receptor tyrosine kinases (RTKs), most notably NTRK1, 2, 3, and others, e.g., ALK, RET, MET and EGFR or intracellular non-receptor tyrosine and serine/threonine kinases such as ABL1 and the RAF proteins, respectively [1,2,3,4]. Fusion genes are the most common abnormality, followed by tandem repeat duplications, point mutations, deletions, and genomic amplifications or overexpression, all resulting in an autoactivation of the kinase domain [4,5,6,7]. Mesenchymal neoplasms driven by activated PKs have a variable histomorphology, but common findings are haphazardly distributed (myo)fibroblastic cells—primitive (more oval) and/or maturated (more elongated)—with monomorphic nuclei, staghorn-like vessels with hyalinization, and the presence of thick collagen bundles. An immunohistochemical hallmark is the co-expression of S100 and CD34, without SOX10 positivity. However, the immunophenotype is also variable [6,8].

Lately, several spindle cell neoplasms with a *GAB1::ABL1* gene fusion as the oncogenic driver have been reported [9,10,11,12,13]. The fusion gene includes the tyrosine kinase domain of *ABL1*, which becomes constitutively active, thereby influencing downstream signaling pathways including MAPK and AKT. Therefore, it seems that these neoplasms are a member of the emerging group of PK-related mesenchymal tumors [2,3,6,10].

In this study, we describe the clinicopathological, genetic, and epigenetic characteristics of five new cases and include a previously reported case [14], adding to our understanding, classification, and identification of these tumors.

## 2. Results

### 2.1. Clinical Characteristics (Table 1)

The six tumors were from three males and three females, aged 7–71 years (median, 17 years; mean, 26 years). They arose in the soft tissue of the neck, axilla, lower back, thigh, and digits (hand and foot). All lesions were excised, with R0 status in five cases, including re-excision in case 2. Case 1 showed microscopically positive margins. Follow-up, available for four patients (cases 2, 4, 5 and 6), was uneventful (1–8 years, median 3 years). The other two lesions (cases 1 and 3) were diagnosed recently.

**Table 1:** Clinical, immunohistochemical, and molecular characteristics of our cases.

Case	Age (y) /Sex	Location/Size	Resection Status/Follow-Up	IHC Pos	IHC Neg	Molecular Features
1	16/m	Neck, im 4 cm	R1, recent case		CD34, S100, EMA, SMA, CKAE1/3, MUC4, STAT6, ALK, pan-TRK	RNA seq: *GAB1::ABL1 (ex6::ex2)* RNA expression classifier: DFSP 0.96 Methylation: DSFP (0.4)-v.12.3; CNV: partial loss chr 10q
2 [14]	7/m	2nd digit foot 2 cm	R1, re-resection R0, NED; 8 y	EMA (weak)	S100, CD34, CKAE1/3, MUC4, SMA, desmin	RNA seq: *GAB1::ABL1 (ex6::ex2)* Methylation: DFSP (0.64)-v.12.3; NTRK-rearranged spindle cell neoplasm (score 0.32)-v13.1, CNV: partial loss chr 2p, complete loss chr 10
3	16/f	Axilla, sc 9.5 cm	R0, recent case	CD34, EMA, GLUT1, (all weak)	S100, SOX10	RNA seq: *GAB1::ABL1 (ex6::ex2)* RNA expression classifier: DFSP score 0.97 Methylation: DFSP (score 0.51)-v12.3; NTRK-rearranged spindle cell neoplasm (score 0.35)-v13.1; CNV: flat
4	26/f	Lower back, sc 2.5 cm	R0, NED; 1.5 y	CD34 (partial), GLUT1 (partial, weak,)	S100, SOX10, CK AE1/3, EMA, MUC4, STAT6	Archer: *GAB1::ABL1 (ex6::ex2)*; Methylation: DFSP (score 0.32)-v12.3; no score > 0.3-v.13.1; CNV: flat
5	71/f	4th digit hand, sc 1.1 cm	R0, NED; 3 y		S100, SOX10, CD34, EMA, CKAE1/3, SMA	Archer: *GAB1::ABL1 (ex6::ex2)* Methylation: DFSP (score 0.5)-v12.3; CNV: focal deletions chr 9q, 15q
6	18/m	Thigh, im 13.5 cm	R0, NED; 1 y	CD34, S100 (partial), GLUT1	EMA, SOX10, SMA, desmin	RNA seq: *GAB1::ABL1 (ex6::ex2)* Methylation: DFSP (score 0.53)-v12.3; NTRK-rearranged spindle cell neoplasm (score 0.67)-v13.1; CNV: gain *EGFR*

Abbreviations: y, years; im, intramuscular; sc, subcutaneous; NED, no evidence of disease; IHC, immunohistochemistry; DFSP, dermatofibrosarcoma protuberans; CNV, copy number variation profile.

### 2.2. Pathological Findings

Grossly, the tumors were homogeneously tan-white and circumscribed. The size ranged from 1.1 to 13.5 cm (median, 3.25 cm; mean, 5.43 cm) (Table 1).

**Figure 1:**
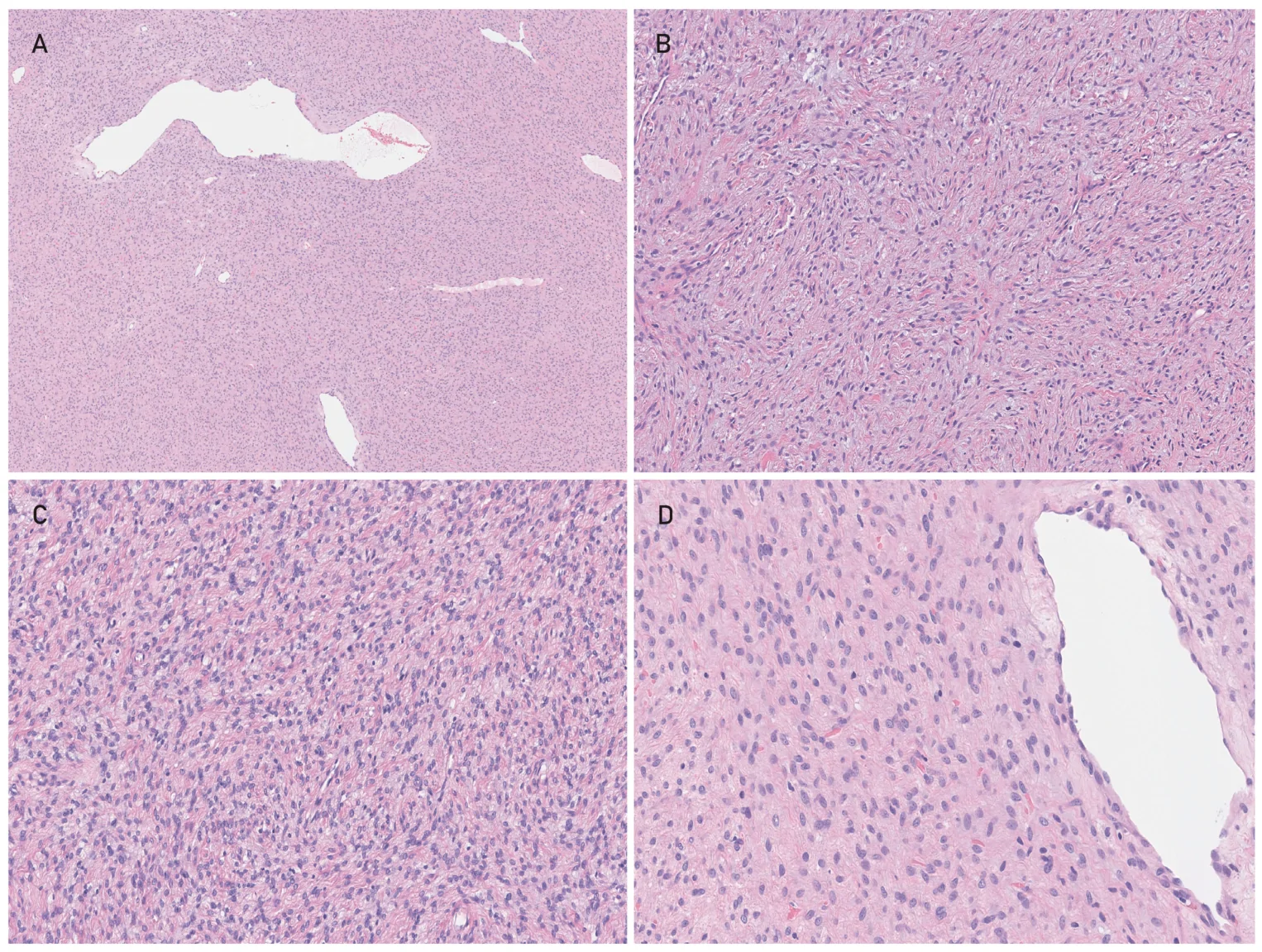
Morphological features (H&E stain). (**A**) Haphazardly arrangend monomorphous spindle cells set in a fibromyxoid stroma with staghorn-like vessels (case 3). Magnification 5×. (**B**) A whorled and storiform growth pattern was seen in some areas (case 2). Magnification 15×. (**C**) The spindle cells possess monomorphic oval and tapered nuclei. Note the collagen bundles and small vessels in the background (case 1). Magnification 15×. (**D**) At higher magnification (25×), nuclei show smooth contours and an evenly distributed chromatin. The cytoplasm is inconspicuous. Mitotic figures were negligible (case 3).

Histologically, all tumors were circumscribed but unencapsulated, with infiltration of the surrounding soft tissue. They were composed of mainly haphazardly arranged monomorphous spindle cells, with some areas showing vague bundles, whorls, or a storiform growth pattern. The nuclei were oval and tapered and had a smooth contour and a homogeneous open chromatin. There was an inconspicuous cytoplasm. Mitotic activity was negligible. The stroma was fibromyxoid with variable wispy or thick collagen bands. The latter were observed in cases 1, 3, 5, and 6. Staghorn-like vessels and hyalinization, also of smaller vessels, was variably observed in all but case 5. None of the lesions showed necrosis (Figure 1).

### 2.3. Immunohistochemical Results (Table 1)

**Figure 2:**
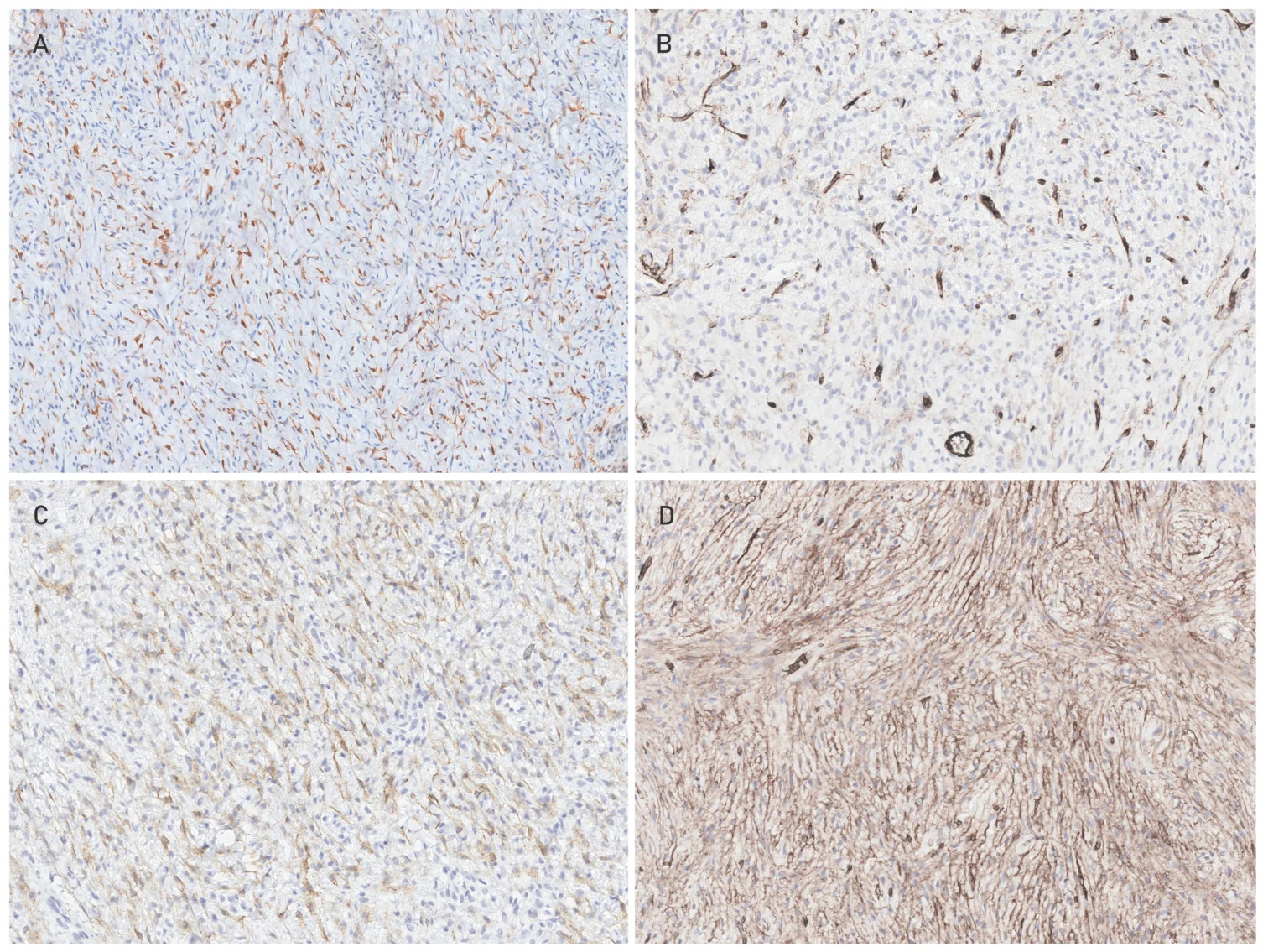
Immunohistochemical results showing variable expression of S100 (case 6) (**A**), CD34 (case 3) (**B**), EMA (case 3) (**C**), and GLUT1 (case 3) (**D**).

CD34 was variably expressed in 3/6 cases, S100 showed a patchy reaction in 1/6 cases and EMA was positive in 2/6 lesions. GLUT1 positivity was seen in all three neoplasms tested (Figure 2). Negative markers were SMA (n = 4), desmin (n = 2), pan-keratin (CKAE1/3) (n = 4), MUC4 (n = 3), SOX10 (n = 4), STAT6 (n = 2), ALK (n = 1), and panTRK (n = 1).

### 2.4. Molecular Results (Table 1)

Using RNA sequencing (n = 4) or anchored multiplex PCR-based targeted RNA sequencing (n = 2), in all cases, a *GAB1 (exon 6)::ABL1 (exon 2)* fusion gene was detected.

In two cases (cases 1 and 3), our RNA expression classifier (version 5.9) was applied, classifying them as dermatofibrosarcoma protuberans (score 0.96).

**Figure 3:**
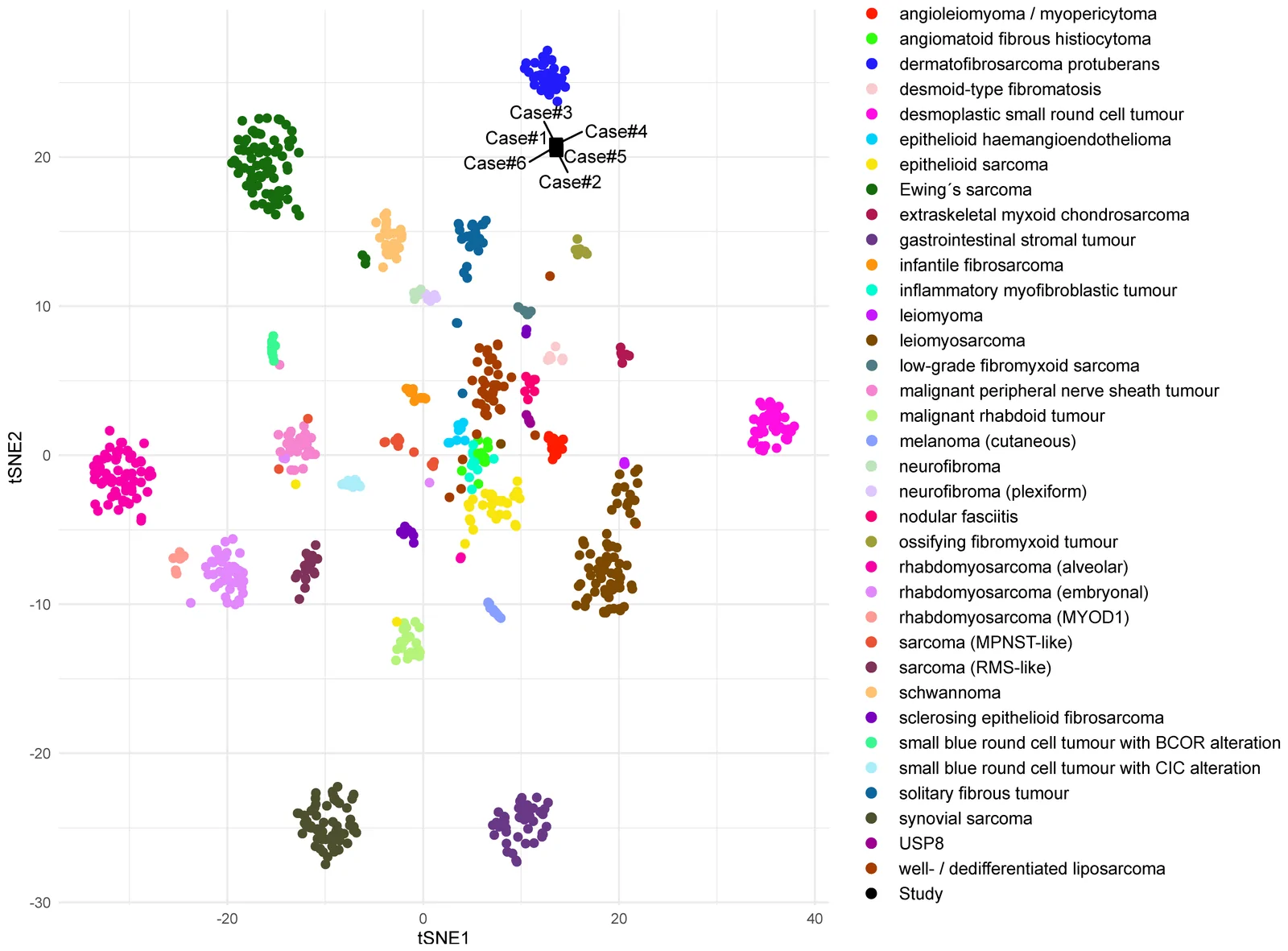
tSNE plot of the study cohort (n = 6, in black) compared to morphological mimics.

Applying the published version of the Heidelberg DNA-methylation sarcoma classifier, all cases showed the highest similarity to the DFSP methylation class, although with low calibrated scores. Using the most recent Heidelberg sarcoma classifier version (v13.1) for diagnostic purposes, 4/6 cases were assigned to the *NTRK*-rearranged spindle cell neoplasm class; however the calibration scores were low and did not support a reliable classification. In the t-SNE analysis, all cases formed a distinct cluster located in close proximity to DFSP (Figure 3).

All cases had relatively stable genomes reflected by their flat CNV profiles; nevertheless, case 1 had partial loss of chromosome 10q, case 2 partial loss of chromosome 2p and complete loss of chromosome 10, focal deletions on chromosomes 9q and 15q were found in case 5, and gain of EGFR was detected in case 6.

## 3. Discussion

The *ABL proto-oncogene 1* (*ABL1*) is named after Herbert Abelson, who isolated, together with Louise Rabstein, a retrovirus that induced B-cell leukemia in murine lymphocytes by a protein with tyrosine kinase activity [15]. The human counterpart was mapped to chromosome 9 and found to be part of the Philadelphia chromosome (chromosome 22), fusing upon a reciprocal translocation (t9;22) (q34;q11) *BCR* and *ABL1* [16,17,18]. This fusion oncogene was primarily attributed to chronic myeloid leukemia (CML)—a major breakthrough in understanding cancer—and later on, to other hematological malignancies [19,20,21,22].

Diverse other *ABL1* fusion partners have been detected in hematological and epithelial malignancies and rarely in mesenchymal tumors with the specific *GAB1::ABL1*, reported so far in 15 benign spindle cell tumors with morphologic similarities with other PK-related mesenchymal tumors [9,10,11,12,13,22,23,24].

The oncogenic *ABL1* fusions lead to constitutive kinase activation by retaining almost the entire ABL1 gene beginning at exon 2, thereby preserving the SH3, SH2, and kinase domain while deleting the N-terminal myristoylated cap that mediates physiological autoinhibition. The consequently disrupted autoinhibition as well as fusion-partner-derived functions, like oligomerization, aberrant subcellular localization, or assembly into signaling complexes that facilitate trans-autophosphorylation and sustained downstream signaling, are thought to be the combined effects. The relative contribution of these mechanisms appears to be fusion-partner-dependent [25]. As the *GAB1::ABL1* fusion product retains the same part of *ABL1*, it is likely that the activating mechanism is similar to other well-studied fusions such as *BCR::ABL1* resulting in activation of ABL1’s downstream pathways including the MAPK, AKT, and JAK/STAT, similar to other PK-related tumors [4,10]. It is not clear why GAB1 (GRB2-associated binding protein1), a multi-substrate docking protein with a prominent role in signal transduction, is the constantly recurrent 5’ fusion partner in these mesenchymal tumors. However, the *GAB1::ABL1* fusion consistently retains exons 1 through 6 of *GAB1*. Therefore, one could hypothesize that the N-terminal PH domain and GRB2-binding region of *GAB1* are under positive selective pressure. Unlike *BCR*, *GAB1* does not contribute a canonical oligomerization motif, raising the possibility that its primary role is to target the fusion kinase to membrane-associated RTK signaling complexes. Given the established role of GAB1 as a central adaptor downstream of MET and other RTKs in fibroblasts and mesenchymal tissues, this architecture may preferentially confer a growth advantage in mesenchymal cells, potentially contributing to the apparent restriction of *GAB1::ABL1* fusions to spindle cell neoplasms [26]. Although this hypothesis remains speculative, it provides a biologically plausible explanation for the pathogenesis of these tumors.

By methylation profiling, all cases formed a cluster in close proximity to the group of PK-related tumors, mainly DFSP as well as spindle cell neoplasms with a *NTRK* rearrangement. In addition, on the RNA level, lesions had a similar expression profile to DFSP. These findings underscore the close relationship of neoplasms with activating alterations in protein kinases, better classified by their driver mutation instead of morphological and immunohistochemical features because of the overlap and treatment modalities based on the activated kinase.

The neoplasms mainly occur in children and young adults, although there is a broad age range. Reported sites are the soft tissue of the extremities (proximal and distal), limb girdles, trunk, and neck [9,10,11,12,13,23,24]. Lesions may arise superficially (subcutaneously) or deeply (intramuscular), and bone erosion has been observed in an individual case [9,10]. Our results are in concert with these findings.

So far, it seems that all reported lesions including ours follow a benign clinical course, with recurrences arising exceptionally [9,10,11,12,13,23,24]. This is in accordance with the flat CNV profile shown here. One patient was treated with imatinib and the tumor showed regression by MRI, which indicates an alternative when surgery is not feasible [10].

From a histomorphological perspective, spindle cell neoplasms with a *GAB1::ABL1* gene fusion show overlap with other PK-related lesions. They are composed of haphazardly arranged bland spindle cells forming vague bundles, storiform formations, and focal whorls within a fibrous to fibromyxoid matrix with staghorn-like and small (branching) vessels. A sclerotic/hyaline background and thick bands of collagen are variably present [9,10,11,12]. Furthermore, lesions show variable expression of S100, CD34, EMA, GLUT1, and claudin. As such, it is not surprising that these neoplasms were previously designated as perineurioma, soft tissue angiofibroma (STAF), or solitary fibrous tumor (SFT) [14,23,24]. However, co-expression of S100 and CD34 without SOX10 positivity is strongly suggestive of a PK-related lesion [2,3,8]. Perineurioma shows characteristic whorls like meningeomas, due to the function of perineurial cells of protective encasement of nerve fascicles, and immunohistochemical expression of EMA, CD34, GLUT1, and claudin [9,23,27]. Soft tissue angiofibroma is most recognizable by its prominent branching vasculature of small vessels, these being far less obvious in *GAB1::ABL1* fused lesions. The immunophenotype of STAF is not specific; however, CD34 and EMA positivity can be misleading, and the detected fusion gene involves *NCOA2* in most of the cases [27]. SFTs commonly do not have the smooth nuclear contours as seen in *GAB1::ABL*-positive cases, and STAT6 is a perfect surrogate marker for the underlying fusion gene [27].

Other differential diagnoses include other PK-related lesions like infantile fibrosarcoma and malignant peripheral nerve sheath tumor (MPNST)-like tumors harboring, as mentioned in the introduction, other hyperactivated kinases like NTRK1,2,3, (B)RAF, MET, RET, and EGFR. Molecular analysis would be relevant for therapeutic purposes, mainly in aggressive lesions [6,28,29].

Other differential diagnoses are low-grade fibromyxoid sarcoma, cellular myofibroma, fat-poor spindle-cell lipoma, desmoid-type fibromatosis, and schwannoma [27,30,31]. All differential diagnoses are listed in Table 2.

In conclusion, spindle cell lesions with a *GAB1::ABL1* fusion activating the tyrosine kinase of ABL1 are a family member within the group of protein-kinase-related mesenchymal neoplasms. Imatinib has already been an effective treatment in an individual case and is therefore a promising therapeutic option for patients with such tumors when surgery is not feasible. However, tumors pursue an indolent clinical course (so far).

## 4. Materials and Methods

The six cases were retrieved from the author’s (referral) files and hematoxilin and eosin (H&E) and immunohistochemical slides were reviewed. Clinical information was available in all cases. Case 2 was already published by Bekers et al. in 2017 [14].

For the Dutch cases, residual tissue was used in accordance with the Dutch Code of Conduct for Responsible Secondary Use of Human Tissue. Patients are informed that leftover tissue may be stored and used for scientific research unless they object (opt-out procedure). The international collaborating centers contributed coded archival material in accordance with their respective institutional and national regulations.

### 4.1. Immunohistochemistry

Sections of four µm thickness were cut from formalin-fixed paraffin-embedded (FFPE) blocks, mounted on pre-coated slides, and dried for at least 10 min at 56 °C. After deparaffinization, the slides were stained using an automated Ventana tissue stainer (BenchMark Ultra, Roche, Tucson, AZ, USA). The following antibodies were used: S100 (ready to use, 4C4.9, Roche), SOX10 (ready to use, EP268, Roche), CD34 (ready to use, QBEND/10, Leica, Wetzlar, Germany), EMA (ready to use, GP1.4, Leica), CKAE1/3 (ready to use, CKAE1/AE3, Leica), GLUT1 (ready to use, polyclonal Cell Marque, Rocklin, CA, USA), SMA (ready to use, Alph-sm-1, Leica), Desmin (ready to use, DE-R-11, Leica), MUC4 (ready to use, 8G7, Cell Marque), STAT6 (ready to use, EP325, Cell Marque), panTRK (1:40, RM423, Sanbio, Uden, The Netherlands), and ALK (ready to use, D5F3, Ventana, Tucson, AZ, USA). Pretreatment was performed according to standard protocols.

### 4.2. Molecular Analyses

#### 4.2.1. Targeted mRNA Sequencing

RNA was isolated from FFPE tissue sections with the Reliaprep FFPE Total RNA Miniprep system (Promega, Madison, WI, USA) according to the manufacturers’ protocol. Up to 250 ng RNA was used for preparing cDNA. Target-enriched NGS libraries were subsequently prepared using the Archer FusionPlex kit (ArcherDX, Coralville, IA, USA) with a custom designed gene panel (Radboudv1) for Illumina, which includes primers for detecting gene fusions upstream of *ABL1* exon 1, 2, 3, 4, 5, and downstream of exon 5 and 6 (RefSeq: NM_005157.6). Sequencing was performed with the NextSeq 550 system (Illumina, San Diego, CA, USA), and data were analyzed using Archer Analysis software (ArcherDX, Boulder, CO, USA, 6.2.7 or 7.3.2).

#### 4.2.2. Whole Transcriptome Sequencing (mRNA Sequencing)

Total RNA was isolated using the AllPrep DNA/RNA/Protein Mini Kit (Qiagen, Hilden, Germany) in keeping with the standard protocol on the QiaCube (Qiagen, Hilden, Germany). For case 6, fresh frozen tissue was used, while for cases 1–5, only FFPE was available. RNA-seq libraries were generated with 300 ng RNA using the KAPA RNA HyperPrep Kit with RiboErase (Roche) and subsequently sequenced on a NovaSeq 6000 system (2 × 150 bp) (Illumina). The RNA sequencing data were processed as per GATK 4.0 best practices workflow for variant calling, using a wdl and Cromwell-based workflow (https://gatk.broadinstitute.org/hc/en-us/sections/360007226651-Best-Practices-Workflows, accessed on 1 June 2026). This included performing quality control with Fastqc (version 0.11.5) to calculate the number of sequencing reads and the insert size Picard (version 2.20.1) for RNA metrics output and MarkDuplicates [33]. The raw sequencing reads were aligned using Star (version 2.7.0f) to GRCh38 and gencode version 29 [34].

#### 4.2.3. DNA Methylation Profiling and Copy Number Variation (CNV) Analyses

DNA was isolated by NorDiag Arrow using the DiaSorin DNA extraction kit (NL) or GeneRead DNA FFPE Kit (Qiagen) according to the respective manufacturer’s instructions. DNA concentration was measured using the Qubit 2.0 fluorometer. Per sample, we used 200 ng (NL) of DNA. Bisulphite conversion was performed with EZ DNA Methylation™ Kit (Zymo Research, Irvine, CA, USA). All methylation data were generated using the Illumina^®^ MethylationEPIC (850 k) or EPICv2 (935K) BeadChip platforms as previously described [35]. Classification of the samples was performed by the Heidelberg sarcoma classifier using both the published version (v12.2) and the most recent version available, v13.1. Further computational analyses were performed employing R version 4.2.0 (https://www.R-project.org, accessed on 1 June 2026). Specifically, raw DNA methylation data were processed using a modified version of the minfi workflow [36] and a t-distributed stochastic neighbor embedding (t-SNE) analysis was carried out using selected potential histologic mimics from a published cohort as a reference. Regarding CNV analyses, the high-density DNA methylation arrays allow for determining CNVs.

## Figures and Tables

**Table 2:** Differential diagnoses [6,27,31,32].

Diagnosis	Morphology	Immuno-Histochemistry	Molecular Features
*GAB1::ABL1* tumors	Haphazardly arranged, vague bundles, whorls, storiform, monomorphic oval or tapered nuclei, HPC-like vasculature	CD34 +/−, S100 +/−, EMA +/−, GLUT1 +/−, claudin +/−	*GAB1::ABL1* fusion gene
Other PK-related tumors	See above (whorls often less obvious)	CD34 +/−, S100 +/−, SMA +/−, desmin −/+, SOX10—and ALK/TRK/BRAF/EGFR + (corresponding to the affected gene)	*NTRK1*,*2*,*3*, *ALK*, *EGFR*, *RET*, *ROS1*, *(B)RAF*, *MET* alterations
Low-grade fibromyxoid sarcoma	Loose fascicles, whorls, uniform spindle cells, tapered to oval nuclei, alternating fibromyxoid matrix, arcades of small vessels	MUC4 +, EMA +, claudin +	*FUS/EWSR1::CREB3L2/CREB3L1* fusion genes
Cellular myofibroma	Biphasic pattern with spindle and ovoid cells, perivascular arrangement, or long fascicles of spindle cells, HPC-like vessels	SMA +, desmin +/−, caldesmon +/−, pancytokeratin +/−	*PDGFRB* mutations *SRF* fusion genes
Desmoid fibromatosis	Long fascicles of slender myofibroblasts, elongated nuclei, small vessels parallel with the bundles, perivascular edema	SMA +/−, desmin +/−, nuclear beta-catenin +	*CTNNB1* or *APC* mutations
Soft tissue angiofibroma	Haphazardly arranged monomorphic spindle cells, delicate branching vasculature	CD34 +/−, EMA +/−	*NCOA2 fusion genes*
Solitary fibrous tumor	Haphazardly arranged monomorphic spindle cells, tapered/angulated to oval nuclei, HPC-like vessels, collagen +/−	CD34 +, STAT6 +	*NAB2::STAT6* fusion genes
Extraneural perineurioma	Whorls, fascicles, storiform pattern, elongated cell processes, oval to tapered/wavy nuclei, fibromyxoid matrix	EMA +, GLUT1 +, claudin +, CD34 +/−	Mutations, deletions of *NF1*, *NF2*
Schwannoma	Mostly encapsulated, variable cellularity, cellular and loose areas, tapered, wavy, (palisading) nuclei, myxohyaline matrix and vasculature	S100 +, SOX10+	Mutations in *NF2*, *SMARCB1*, *LZTR1*, *LATS*, *ARID1*, *DDR*, *SOX10*

Abbreviations: HPC; hemangiopericytoma.

## Data Availability

The original contributions presented in this study are included in the article. Further inquiries can be directed to the corresponding author(s).
